# Systemic analysis of urinary stones from the Northern, Eastern, Central, Southern and Southwest China by a multi-center study

**DOI:** 10.1186/s12894-018-0428-2

**Published:** 2018-12-13

**Authors:** Rui-hong Ma, Xiao-bing Luo, Qin Li, Hai-qiang Zhong

**Affiliations:** 1 The Department of Clinical Laboratory, The Sixth People’s Hospital of Nansha, Xingye Road No. 7, Dagang Town, Nansha, Guangzhou, 511470 People’s Republic of China; 2 The Department of Pulmonary, Critical Care and Sleep, Yale School of Medicine, New Haven, USA

**Keywords:** Urinary stones, Fourier transform infrared spectroscopy, Scanning electron microscopy, X-ray energy spectrometer, Calcium oxalate stones, Uric acid stones, Regional distribution

## Abstract

**Background:**

To provide some basis for the prevention of urinary stones in general population, we did a systemic analysis of urinary stones from Northern, Eastern, Central, Southern and Southwest China by a multi-center study.

**Methods:**

A total of 11,157 urinary stones from Northern, Eastern, Central, Southern and Southwest China were obtained and analyzed by Fourier transform infrared spectroscopy. Combined with scanning electron microscopy and X-ray energy spectrometer, urinary stones were classified into different types. Furthermore, the correlation between stone types and clinical characteristics, as well as their regional distribution were elucidated.

**Results:**

Calcium oxalate stones were the most common type in each region, followed by calcium oxalate-calcium phosphate mixed stones, uric acid stones and calcium phosphate stones. The distribution of calcium oxalate stones were highest prevalence in Southwest China (67.9%, *P < 0.05*), followed by Eastern and Northern China. Anhydrous uric acid stones, with a constituent ratio of 19.3% in Southern China, and 13.7% in Central China, were significantly higher than that in other regions (*P < 0.05*). Elements analysis indicated varieties among stone types as well as distribution regions. Moreover, the clinical characteristics were highly correlated with stone types and anatomical locations but not their distribution regions.

**Conclusions:**

The material and elements composition of urinary stones among different regions showed some varieties. Calcium oxalate stone has the highest constituent ratio in Southwest China, while anhydrous uric acid stone has the highest constituent ratio in Southern China. Moreover, the clinical characteristics were highly correlated with stone types and anatomical locations but not their distribution regions.

## Background

Urinary stone disease, also known as urolithiasis, is one of the leading afflictions worldwide. The incidence differs with geographic distribution. In general, as described in detail previously [1, 2], the average prevalence is higher in western countries (5–9% in Europe, and 13% in North America) than the eastern (1–5% in Asia). While in Saudi Arabia, nearly 20% people suffer from it. In China, the disease approximately affects 4% of population and increases steadily during the recent 20 years, due to high calories intake from dietary and lack of exercise in lifestyle [3–5]. Therefore, effective prevention strategy and precise treatment are needed to alleviate the burden caused by high prevalence of urolithiasis.

Urolithiasis cause undoubtedly considerable burden on public health worldwide not only by its high prevalence but also for the recurrence rate. A report in Germany indicated that about 40% of patients suffered from recurrent urolithiasis once or more, and even over 10% underwent five or more stone episodes [6]. Therefore, it is important to clearly define the etiology and establish an effective prevention program on urinary stones [7]. As the composition of urinary stone may provide some clue for the formation process, stone analysis is important in determining the possible etiology of urinary stones. Moreover, there exists a wide geographic variation in proportion for different stone types. The phenomenon is demonstrated well in the United States but still limited in China [8, 9]. Therefore, in the present study, we did a systemic analysis of urinary stones from Northern, Eastern, Central, Southern and Southwest China by a multi-center study to describe the commons and differences of stone composition, major and trace elements as well as clinical characteristics.

## Methods

### Ethics statement

The sample collection procedures were explained to all patients. Written informed consent was obtained from all patients. The principles outlined in the Declaration of Helsinki of 1975 (revised in 1983 and 1989) were followed throughout the study period. The study was approved by both the Ethics Committee of The Sixth People’s Hospital of Nansha, Guangzhou (reference number is No: 20130821057P) and the Ethics Committee of The Kingmed Diagnostics Center of Guangzhou (KM20130149).

### Subjects and specimens

Urinary stones from 11,157 urolithiasis patients were collected from department of the urinary surgery in The Sixth People’s Hospital of Nansha, Guangzhou, and the Kingmed Diagnostics Center of Guangzhou during September 2013 to September 2017. The patients consisted of 7437 males, ranged from 18 to 95 years old, the mean age was 49.61 ± 14.40 (*Mean* ± *S.D*) years, and 3720 females, ranged from 20 to 88 years old, the mean age was 49.31 ± 13.23 (*Mean* ± *S.D*) years. The age of the two groups had no statistically significant difference.

### China’s regional division

Northern China includes: Beijing, Tianjin, Hebei, Shanxi, Inner Mongolia provinces; Eastern China includes: Shanghai, Shandong, Jiangsu, Anhui, Jiangxi, Zhejiang, Fujian provinces; Central China includes: Hubei, Hunan, Henan; Southern China includes: Guangdong, Guangxi, Hainan provinces; Southwest China includes: Chongqing, Sichuan, Guizhou, Yunnan, Tibet provinces.

### The recruitment of the urinary lithiasis patients

Of the above patients, 844 from Northern China, including 646 males, with average age of 45.58 ± 13.45, and 198 females, with average age of 43.16 ± 14.87; 2149 from Eastern China, including 1277 males, with average age of 47.07 ± 13.19, and 872 females, with average age of 46.95 ± 12.56; 713 from Central China, including 463 males, with average age of 49.65 ± 14.36, and 250 females, with average age of 48.98 ± 13.11; 6423 from Southern China, including 4339 males, with the average of 50.50 ± 14.75, and 2084 females, with the average age of 50.31 ± 13.38; 1028 from Southwest China, including 712 males, with the average of 46.85 ± 14.00, and 316 females, with the average age of 47.92 ± 13.09. The proportion of males and females from each region is slightly different, with a higher proportion of males in Northern China, and a lower in Eastern China. The age has some differences among different regions, with older age in Southern China and Central China, and younger age in Northern China.

### Systemic classification of urinary stones

A total of 11,157 urinary stones were classified into different types with systemic classification combing using FTIR spectroscopy, SEM and X-ray energy spectrometer, the procedure has been detailed in our previous work [7].

### Comparison of stone composition and clinical characteristics in patients with different regions

Urinary lithiasis patients were divided into five groups according to the regions, such as Northern China, Eastern China, Central China, Southern China and Southwest China, and urinary stones were divided into five groups according to the composition, such as calcium oxalate stones, calcium phosphate stones, uric acid stones, calcium oxalate-calcium phosphate mixed stones and other kinds of stone group. Others consisted of magnesium ammonium phosphate, cystine, brushite, urate stones and some subtypes of mixed stones that were minority. Stone composition and clinical characteristics in different regions were compared and analyzed.

### Statistic methods

Age was analyzed using One-Way ANOVA and presented as mean ± SD, while the ratio of male and female, and the ratio with different types of stones as well as from different anatomical locations or regions were analyzed using a chi square test. LSD and the partitions of the chi square methods were used for multiple comparisons using IBM SPSS Statistics 24 software. *P* < 0.05 was regarded as statistically significant.

## Results

### Urinary stone composition were distinct in different regions of China

Calcium oxalate (whewellite and weddellite) stones were the most common in each region, followed by calcium oxalate-calcium phosphate mixed, anhydrous uric acid and calcium phosphate stones. The distribution of calcium oxalate stones were highest prevalence in Southwest China (67.9%, *P < 0.05*), followed by Eastern and Northern China. Anhydrous uric acid stones, with a constituent ratio of 19.3% in Southern China, and 13.7% in Central China, were significantly higher than that in Northern, Eastern and Southwest China (*P < 0.05*). The constituent ratio of calcium phosphate stones (about 5%) among different regions had no statistical differences (*P > 0.05*). The stone type constitution in the five regions of China was shown in Table 1.

**Table 1:** Distribution of stone types in five regions of China

Stone type/regions	Calcium oxalate	Calcium phosphate	Uric acid	Calcium oxalate-calcium phosphate mixed	Other types	Total
Northern	485 (57.5%)	42 (5.0%)	52 (6.2%)	183 (21.7%)	82 (9.7%)	844 (100%)
Eastern	1360 (63.3%)	121 (5.6%)	123 (5.7%)	490 (22.8%)	55 (2.6%)	2149 (100%)
Central	377 (52.9%)	36 (5.0%)	98 (13.7%)	177 (24.8%)	25 (3.5%)	713 (100%)
Southern	3317 (51.6%)	359 (5.6%)	1242 (19.3%)	1221 (19.0%)	284 (4.4%)	6423 (100%)
Southwest	698 (67.9%)	48 (4.7%)	60 (5.8%)	188 (18.3%)	34 (3.3%)	1028 (100%)
Total	6237 (55.9%)	606 (5.4%)	1575 (14.1%)	2259 (20.2%)	480 (4.3%)	11,157 (100%)

### Element composition and distribution in each type of urinary stone

**Fig. 1:**
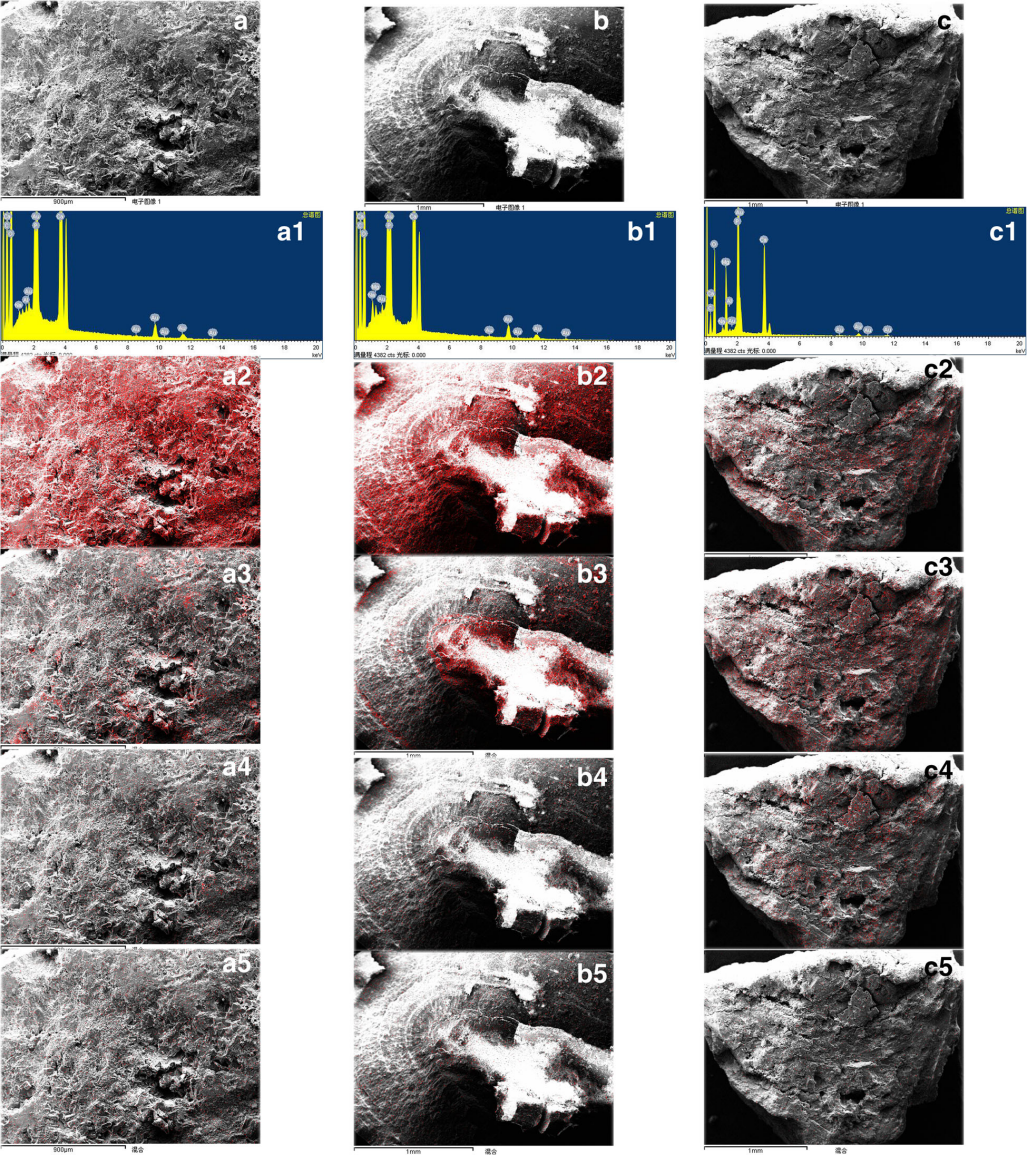
Element distribution in general stones (the red dot represented element distribution). **a** calcium oxalate stones with micro dishes of calcium phosphate: calcium oxalate crystals were distributed in the outer layer and profile, with micro dishes of calcium phosphate crystals in the core. A1.The energy spectrum, A2. Calcium distribution, A3. Phosphorus distribution, A4. Magnesium distribution, A5. Natrium distribution; **b** calcium oxalate-calcium phosphate mixed stones: calcium oxalate crystals were distributed in the outer layer, calcium phosphate crystals were in the core. B1. The energy spectrum, B2. Calcium distribution, B3. Phosphorus distribution, B4. Aluminum distribution, B5. Natrium distribution; **c** Calcium phosphate stones: a small amount of ammonium magnesium phosphate crystals were scattered in the profile. C1. The energy spectrum, C2. Calcium distribution, C3. Phosphorus distribution, C4. Magnesium distribution, C5. Aluminum distribution

**Fig. 2:**
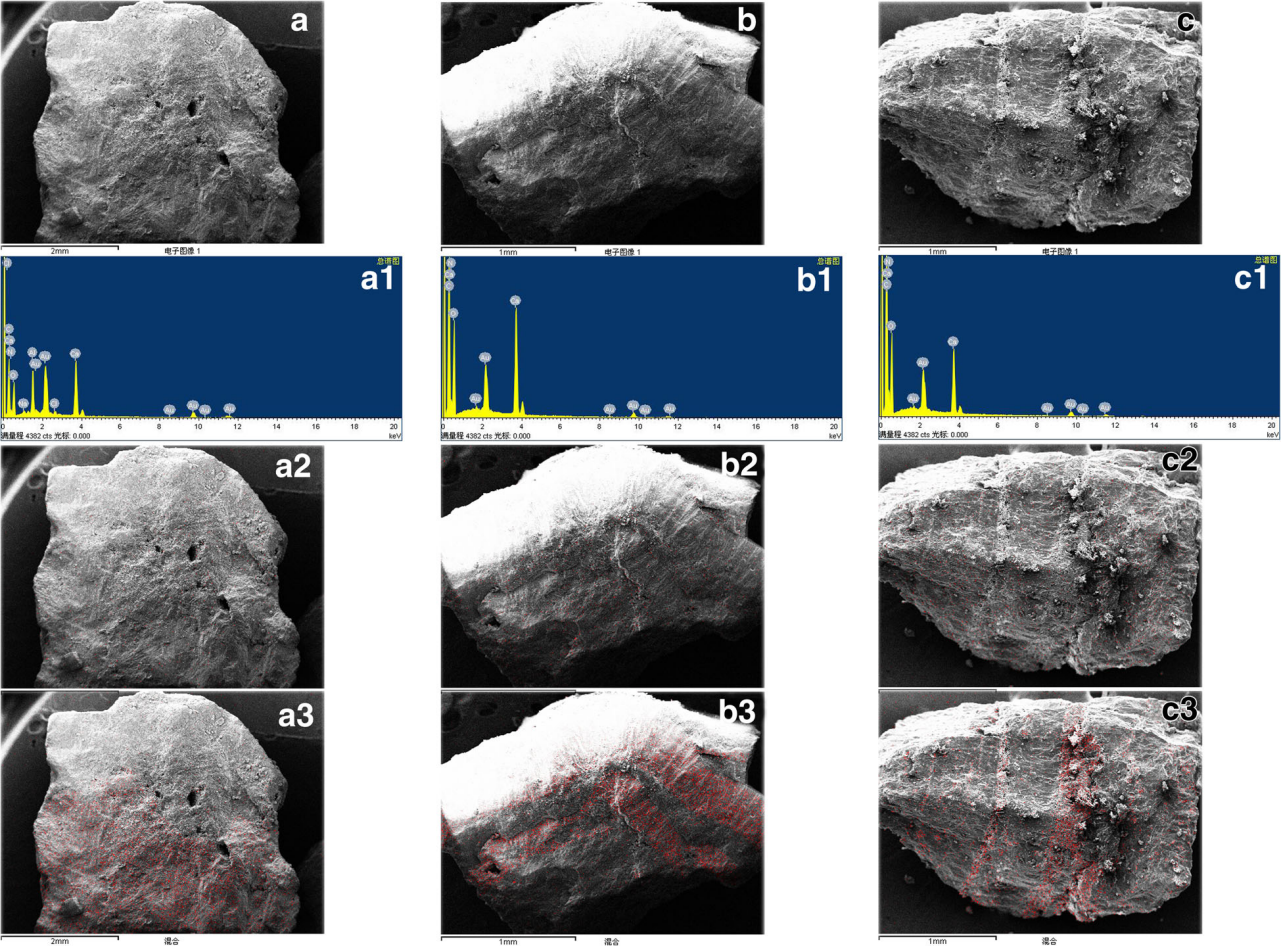
Element distribution in general stones: uric acid stones mixed with some calcium oxalate crystals (the red dot represented element distribution). **a** Uric acid- calcium oxalate mixed stones: uric acid crystals were distributed in the outer layer, and calcium oxalate crystals in the core. A1. The energy spectrum, A2. Nitrogen distribution, A3. Calcium distribution; **b** Calcium oxalate - uric acid mixed stones: calcium oxalate crystals were distributed in the outer layer, and uric acid crystals in the core. B1. The energy spectrum, B2. Nitrogen distribution, B3. Calcium distribution; **c** Uric acid stones mixed with micro dishes of calcium oxalate crystals: uric acid crystals were distributed in circular layer, and calcium oxalate crystals were scattered between the layers: C1. The energy spectrum, C2. Nitrogen distribution, C3. Calcium distribution

**Fig. 3:**
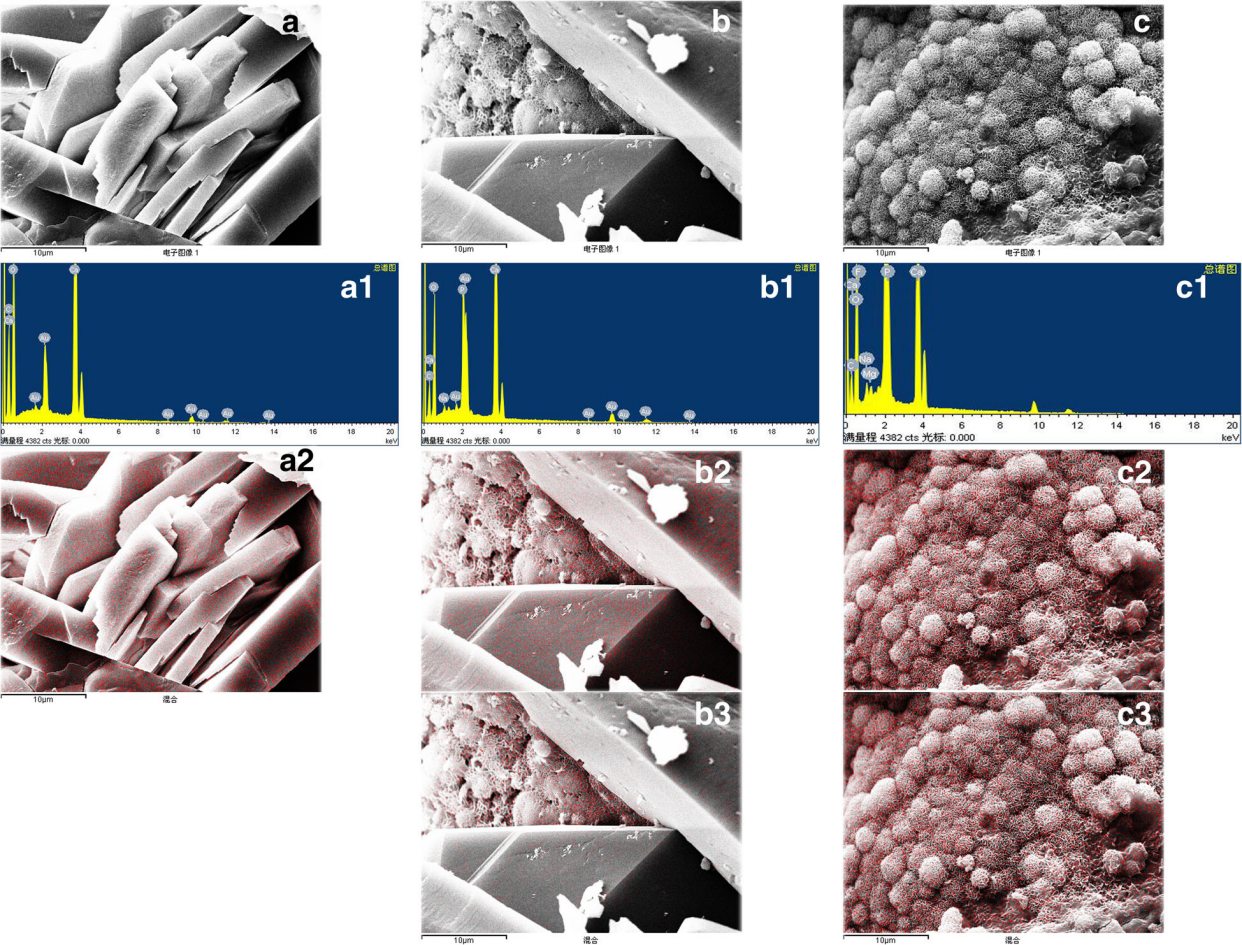
Calcium and phosphorus distribution in calcium oxalate and calcium phosphate crystals (the red dot represented element distribution). **a** calcium oxalate crystals: A1. The energy spectrum, A2. Calcium distribution; **b** Calcium oxalate- calcium phosphate mixed crystals: B1. The energy spectrum, B2. Calcium distribution, B3. Phosphorus distribution; **c** Calcium phosphate crystals: C1. The energy spectrum, C2. Calcium distribution, C3. Phosphorus distribution

**Fig. 4:**
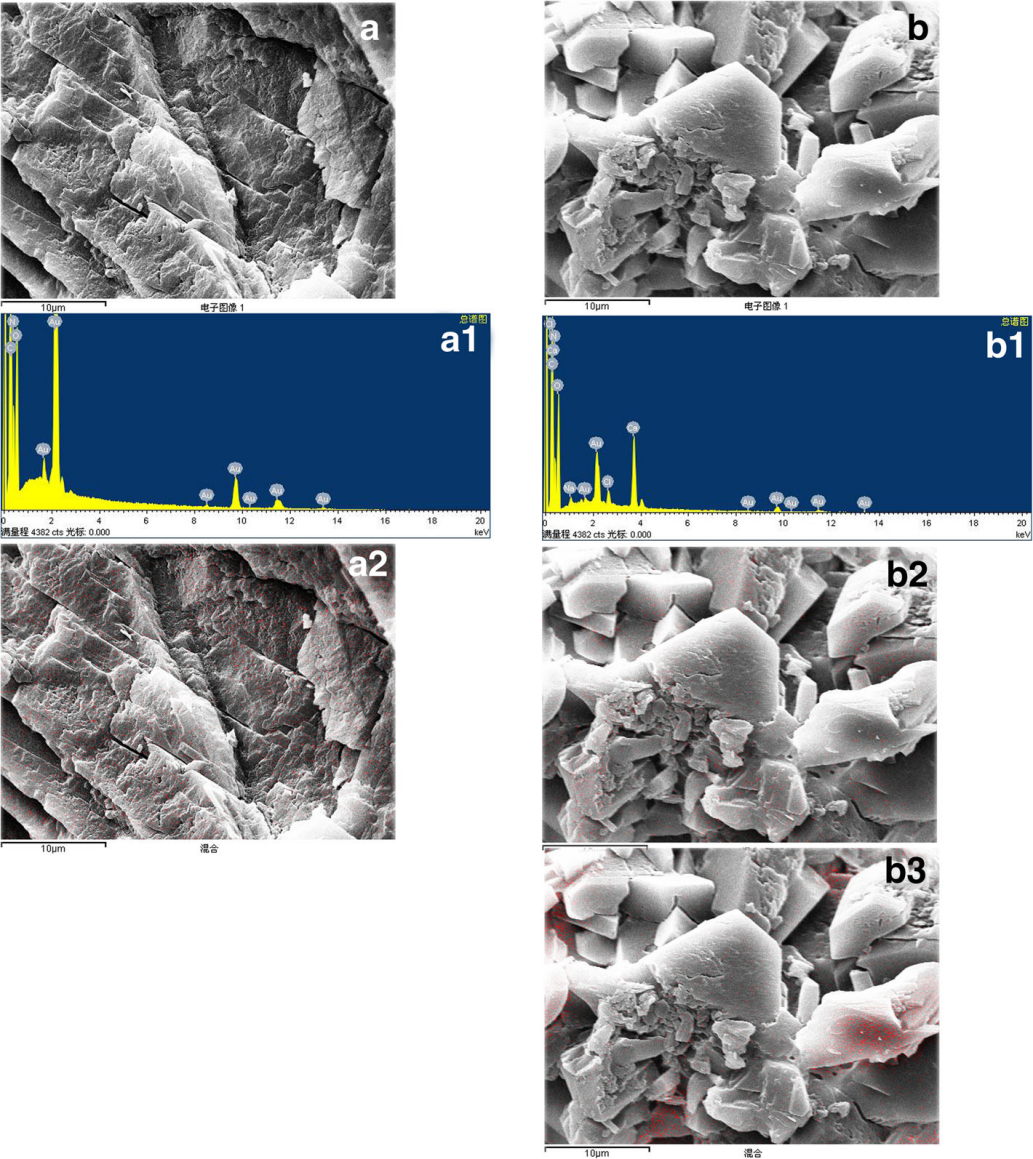
Nitrogen and calcium distribution in uric acid and uric acid- calcium oxalate mixed crystals (the red dot represented element distribution). **a** Uric acid crystals: A1. The energy spectrum, A2. Nitrogen distribution; **b** Uric acid- calcium oxalate mixed crystals: B1. The energy spectrum, B2. Nitrogen distribution, B3. Calcium distribution

Besides the main elements (Italic), each type of stone still contains a small amount of metal and non-metallic elements, which had some varieties among stone types as well as regions. For calcium oxalate stones, besides the main elements carbon, oxygen, calcium, they still contain some sodium, kalium, magnesium, chlorine, aluminum, phosphorus, niobium, hafnium, sulphur, etc. For calcium phosphate stones, in addition to carbon, oxygen, calcium, phosphorus, they still contain some sodium, kalium, magnesium, chlorine, aluminium, niobium, fluorine, zinc, technetium, hafnium, chrome, etc.; and for those anhydrous uric acid stones, in addition to carbon, oxygen, nitrogen, they still had some sodium, calcium, aluminium, chlorine. Urinary stones from Southern China usually contained some zinc, and those from Southwest China usually contained some silicon, which seems more complex and diverse compared with other regions (Table 2). Mapping analysis of element distribution in general stones with X-ray energy spectrometer showed that for those calcium oxalate stones with micro dishes of calcium phosphate and calcium oxalate-calcium phosphate mixed stones, calcium oxalate crystals were always distributed in the outer layer while calcium phosphate crystals were in the core (Fig. 1a, b). Moreover, for those calcium phosphate stones, usually with a small amount of ammonium magnesium phosphate crystals scattered in the profile (Fig. 1c). Uric acid stones, mixed with micro or macro dishes of calcium oxalate crystals, displayed inconsistent crystal distribution characteristics. In some of the uric acid stones, uric acid crystals were shown in the outer layer and calcium oxalate crystals were in the core. Calcium oxalate crystals in the outer layer while uric acid crystals in the center were also found in some kind of uric acid stones. Besides, uric acid crystals mixed with calcium oxalate crystals in circular layers were observed in uric acid stones (Fig. 2). Mapping analysis of element distribution in micro field with magnification of 3000 times showed that there was only calcium distribution in calcium oxalate crystals, and both calcium and phosphorus distribution in calcium phosphate crystals and calcium oxalate-calcium phosphate mixed crystals, with a consistent distribution in calcium phosphate crystals and inconsistent distribution in calcium oxalate-calcium phosphate mixed crystals. Calcium was distributed in both calcium oxalate and calcium phosphate crystals; phosphorus was distributed in calcium phosphate crystals (Fig. 3). Moreover, there was only nitrogen distribution in uric acid crystals, and both nitrogen and calcium distribution in uric acid-calcium oxalate mixed crystals (Fig. 4).

**Table 2:** Element composition in each type of urinary stone with different regions

Stone type/region	Calcium Oxalate	Calcium Phosphate	Uric Acid	Calcium Oxalate-Calcium Phosphate Mixed
Northern	*C*, *O, Ca*, Na,Cl,Mg,Al,P,Nb,Tc	*C, O, Ca, P,* Na,Mg,F,Cl,Nb,Tc,Hf,Cr	*C, O, N,* Ca	*C, O, Ca, P, *Na,Mg,K,F,Cl,Al,Nb
Eastern	*C, O, Ca*,Na,Cl,Mg,Al,P,K,Nb,Tc,Br	*C, O, Ca, P, *Na,Mg,K,Al,Cl,Nb,Tc,Hf	*C, O, N,*Na,Al,Cl,Ca	*C, O, Ca, P, *Na,Mg,K,F,Cl,Al,Nb,Tc,Hf,Fe
Central	*C, O, Ca*,Na,Al, Mg,P	*C, O, Ca, P, *Na,Mg,Nb	*C, O, N,*Ca	*C, O, Ca, P,* Na,Mg,F,Cl,Nb,Tc
Southern	*C, O, Ca,*Na,Cl,Mg,Al,P,K,F,Si,Nb,Tc,Hf,Zn	*C, O, Ca, P, *Na,Mg,Nb, Tc,Hf, Zn,F	*C, O, N,*Al,Ca	*C, O, Ca, P,* Na,Mg,F,Cl,Al,Nb,Tc,Hf,Zn
Southwest	*C, O, Ca, *Na,Cl,Mg,Al,P,K,Nb,Tc,Zn.S,Fe,Si	*C, O, Ca, P,* Na,Mg,K,F,Cl, Nb, Tc,Si	*C, O, N,*Al,Cl,Ca,Si	*C, O, Ca, P, *Na,Mg,F,Cl,Al,Nb, Tc,Si

The italics are the main elements of corresponding stone types

### The clinical characteristics of each type of urinary stone

Urinary stones were divided as upper urinary stones (kidney and ureteral stones) and lower urinary stones (bladder and urethral stones) according to the anatomical location. In our current studies, we did correlation analysis on 9073 urinary stones and clinical characteristics of these patients. Interestingly, the clinical characteristics were highly correlated with stone types and anatomical locations but not their regions. As shown in Table 3, 7448 cases were upper urinary stones while 1625 were lower urinary stones, and the ratio were 4.6:1. Among the patients with upper urinary stones, 4523 were males, accounting for 60.7%, while those with lower urinary stones, 1504 were males, accounting for 92.6%, which had statistically significant difference (*P < 0.05*). It was indicated in the present study that calcium oxalate stone was the main stone type of upper urinary stones, constituting 60.6%, while uric acid stone was that of lower urinary stones, constituting 43.0% (*P < 0.05*). Patients with calcium oxalate stones were mainly males between the ages of 30 and 60, accounting for 49.4%, while those with calcium phosphate stones were mainly females between the ages of 30 and 60, accounting for 42.1%, and those with uric acid stones were mainly elder males over the age of 60, accounting for 45.8%, the difference was statistically significant (*P < 0.05*). Calcium phosphate stones and calcium oxalate-calcium phosphate mixed stones were mainly from upper urinary tract. Patients with calcium oxalate-calcium phosphate mixed stones were males or females, the ratio tend to 1, while those with magnesium ammonium phosphate and urate stones were mainly females, and those with brushite and cystine stones were mainly males (not list in the table).

**Table 3:** Clinical characteristics of patients with each type of stone

Stone type		Calcium Oxalate		Calcium Phosphate		Uric Acid		Calcium oxalate-calcium phosphate mixed		Other rare type		Total
	No	4995		489		1398		1831		360		9073
	Rate (%)	55.05%		5.39%		15.41%		20.18%		3.97%		100%
	Age (years) & gender	Male	Female	Male	Female	Male	Female	Male	Female	Male	Female	
Upper urinary stones	< 20	48	18	0	6	0	0	12	6	0	6	96
	20~29	174	87	18	33	2	2	105	54	18	2	495
	30~39	546	204	18	71	46	8	213	120	21	15	1262
	40~49	909	432	24	60	132	40	258	225	48	29	2157
	50~59	753	522	51	62	168	80	183	189	42	48	2098
	60~69	396	273	24	29	95	78	72	75	15	33	1090
	> = 70	99	57	0	6	25	23	2	23	6	9	250
	Total	2925	1593	135	267	468	231	845	692	150	142	7448
Lower urinary stones	< 20	3	3	3	0	3	2	0	0	2	3	19
	20~29	3	3	0	1	10	1	42	12	0	0	72
	30~39	45	1	15	9	15	3	27	6	0	3	124
	40~49	87	2	9	1	33	3	36	3	9	3	186
	50~59	126	6	15	3	78	23	60	3	6	3	323
	60~69	108	3	15	1	205	3	54	2	12	3	406
	> = 70	87	0	12	3	315	5	48	1	21	3	495
	Total	459	18	69	18	659	40	267	27	50	18	1625
Total		3384	1611	204	285	1127	271	1112	719	200	160	9073

## Discussion

Urolithiasis, a common urological disease with multiple etiologies, has been a public burden to the world. In the last few decades, urolithiasis has been steadily increased [6, 10]. The present data indicated that the highest incidence of urinary stones occurred in 40–49 years old males and 50–59 years old females respectively. Urinary stones can be classified as metabolic stones (calcium oxalate and some uric acid or urate) and infectious stones (struvite, apatite or a mixture of the two), males are more likely to have metabolic stones, while females, with a high probability of urinary tract infections than men, are more prone to infectious stones [11]. The present data showed that patients with calcium oxalate stones and uric acid stones were mainly males, while those with calcium phosphate stones and magnesium ammonium phosphate (struvite) stones were mainly females.

The present data showed calcium oxalate stone was the most frequent stone type in all age groups, with high frequency between the ages of 30 and 60 years, and relatively low frequency in the younger and elderly. As previously detailed [12], the intake of oxalate and calcium in the diet has an important effect on urinary oxalate excretion, which also play a major role in the formation of calcium oxalate stone. Increased intake of dietary oxalate lead to an increase of urinary oxalate excretion, and decreasing dietary oxalate lowers urinary oxalate excretion [13]. Dietary calcium has a bidirectional effect on urinary oxalate excretion, Borghi’s study showed the low-calcium (10 mmol/day) group presented a higher relapse of calcium oxalate stones than the normal-calcium (30 mmol/day) group [14]. The high percentage of calcium oxalate stone in Southwest China may be originated from water quality, soil and the local dietary structure. It was supposed that the major contributing factor maybe the increase of Ca^2+^/Mg^2+^ ratio (in meq) in drinking water [15]. Additionally, the high calcium-content in local plants grown on karst soils along with the high ingestion of oxalate food may lead to the high incidence of calcium oxalate stones [15]. In China, karst soils mainly distributes in Southwest of China (Chongqing, Sichuan, Guizhou, Yunnan) [16], with a consistent distribution with calcium oxalate stones, which presents the highest constituent ratio in the Southwest among all areas of China. Meanwhile, calcium oxalate- calcium phosphate mixed stones account for a large proportion. In our opinion, it is difficult to manage urinary stones as stone recurrence is common because residual stone fragment contain bacteria and become the core of recurrent stone [11]. Furthermore, a previous research showed urinary tract infection was closely related to the existence of amorphous carbonated calcium phosphate (or whitlockite) and carbonated apatite [17]. Other research indicated that for recurrent stones, the admixed CaOx/CaPO_4_ stones were converted from pure CaPO_4_ more commonly than from pure CaOx stone [18]. Therefore, we conclude that calcium oxalate-calcium phosphate mixed or calcium oxalate stones with CaPO_4_ in the core, the infectious factors maybe first involved in the stone process, and a considerable proportion of which may be recurrent stones.

Uric acid stones has a prevalence of about 10% among all stones [19], but this percentage become extremely high in those patients with gout [20]. Consistent with other reports [21], we found that uric acid stones increased with older age, elder males over the age of 60 accounted for 45.8% of total uric acid stones in the present study. UA urolithiasis is a complex disease that is affected by many factors. Diseases relevant with aging, such as obesity, insulin resistance and diabetes, are also associated with low urinary pH and UA stone formation [22–24]. Furthermore, as previously described [25], persistent acidic urine (pH ≤ 5.5) is considered to be the most important risk factor for the formation of UA stones. Additionally, environmental factors may have a prominent influence on the composition of urinary stones. As reported [26], climate and diet play a crucial role in the formation of UA stones. Hot and dry climate increases fluid losses with reduced urinary volume and decreased urinary pH. Consistent with these, our studies showed the incidence of UA stone in Southern China was the highest, followed by Central, Northern, Eastern and Southwest in China. For those uric acid-calcium oxalate mixed stones, some research indicated that patients with this kind of mixed stones presented with metabolic abnormalities that promote both CaOx and UA stone formation [27, 28]. We supposed that the process of uric acid crystals deposition and that of calcium oxalate crystals deposition may promote each other, whichever occurred first, the formation process probably accelerate when the other emerged. Therefore, uric acid and calcium oxalate crystals may be distributed in the core or outer layer or in circular layers.

There were other epidemiologic surveys in other countries. A study in the United States indicated that the prevalence of urinary stones in Southeast was almost twice of that in Northwest, which was ascribed to climatologic factors [29]. Moreover, another research based on five U.S. metropolitan areas further confirmed the strongest association between high temperature and kidney stones [30]. Furthermore, a multi-center analysis in Germany also showed significant regional differences in urinary stone distribution: a higher rate of uric acid stones in Southern Germany was supposed to be associated with their diets based on more red meat, and the higher incidence of infectious stones in Eastern Germany maybe due to lower standard medical care and infections [19].

As reported, urinary stone form in multiphase steps, apart from the specific conditions like molar concentrations, pH, temperature, etc., different types of trace elements may also affect the crystallization process [31]. In the present study, X-ray energy spectrometer analysis indicated that besides the major elements of urinary stones, other minor elements, such as Na, Cl, Mg, Al, K, Fe, Zn, S, F, Nb, Tc, Hf, Cr and Si were also detected. According to the present study and the previous reports, we found they might play an important part in the urinary stone formation. What is the role of different elements? A study showed sodium concentration was marginally associated with increased risk of stone formation in elder participants (mean age of 61 years) with a history of kidney stone [32]. Nevertheless, the relationship between the sodium content and the risk of formation urinary stone is still under limited studies. Magnesium is also essential to stone formation. One study suggested an oral of 500 mg magnesium oxide each day may inhibit the crystallization of CaO_X_ stones in normal volunteers [33], yet a research on rats implied that strict restriction of dietary magnesium may promote stone formation [34]. Potassium was also known as a lithogenesis inhibitor, a randomized trial demonstrated that potassium citrate can prevent the formation of new stones in idiopathic hypocitraturic calcium nephrolithiasis patients [35]. Aluminum is a non-essential element for human. One study showed that a positive correlation between aluminum level in the urine and stones, as well as between urine and hair, suggesting aluminum probably play a role in stone formation [36]. Another study indicated that Al^3+^ affected crystal growth of calcium phosphate at physiologic concentration, but evidence for the inhibitory role in calcium phosphate stone growth was insufficient [37]. Sulphur is an essential nonmetal element existing in all the cells of human body, and mainly obtained from sulfur-containing proteins in diet. The effects of sulphur on urinary stone formation are almost unknown, but yet a report indicated animal protein-induced hypercalciuria result in the presence of nonresorbable calcium sulfate in the tubular lumen, which may further lead to sulfate production and stone formation [38]. Moreover, the effects of zinc on urinary stone formation was controversial. One research suggested a positive correlation of dietary zinc intake and the risk of kidney stone [39], another showed zinc acts as an inhibitor on the formation of calcium oxalate stone [40]. Silicon may be an essential element for human. A previous research showed silicon plays an essential part in the metabolism of calcium and magnesium [41]. Furthermore, another research indicated ingestion of trisilicate may result in the formation stone entirely or partially composed of silica [42]. Nevertheless, how silicon make an effect on urinary stone formation is still unclear. In the present study, sodium, magnesium, potassium presented a scattered distribution in the profile of urinary stones, while aluminum was more concentrated in the interior than the crust of the stones and usually accompanied with calcium phosphate crystals, which suggest that aluminum may play a role in the early phase of lithogenesis.

## Conclusions

The systemic analysis of urinary stones from the Northern, Eastern, Central, Southern and Southwest China by a multi-center study indicated that the material and elements composition of urinary stones among different regions had some difference. Calcium oxalate stone has the highest constituent ratio in Southwest China, while anhydrous uric acid stones have a highest constituent ratio in Southern China. It was confirmed that composition of urinary stones has wide geographic variation and the formation of which was associated with environmental factors (water quality and soil) as well as dietary structure, weather and climate. Moreover, the clinical characteristics were highly correlated with stone types and anatomical locations but not their regions. Furthermore, different type of urinary stones presented distinct elemental composition and distribution, and some minor element may play an important role in the formation of urinary stones. These findings would be meaningful to prevent stone occurrence and recurrence in general population from different areas of China in future.

## Abbreviations

CaOx: Calcium oxalate
FTIR: Fourier transform infrared spectroscopy
SEM: Scanning electron microscopy
UA: Uric acid
